# Shedding of TRAP by a Rhomboid Protease from the Malaria Sporozoite Surface Is Essential for Gliding Motility and Sporozoite Infectivity

**DOI:** 10.1371/journal.ppat.1002725

**Published:** 2012-07-26

**Authors:** Ijeoma Ejigiri, Daniel R. T. Ragheb, Paco Pino, Alida Coppi, Brandy Lee Bennett, Dominique Soldati-Favre, Photini Sinnis

**Affiliations:** 1 Department of Microbiology, New York University School of Medicine, New York, New York, United States of America; 2 Department of Molecular Microbiology and Immunology, Johns Hopkins Bloomberg School of Public Health, Baltimore, Maryland, United States of America; 3 Department of Microbiology, School of Medicine, University of Geneva, Geneva, Switzerland; Faculdade de Medicina da Universidade de Lisboa, Portugal

## Abstract

*Plasmodium* sporozoites, the infective stage of the malaria parasite, move by gliding motility, a unique form of locomotion required for tissue migration and host cell invasion. TRAP, a transmembrane protein with extracellular adhesive domains and a cytoplasmic tail linked to the actomyosin motor, is central to this process. Forward movement is achieved when TRAP, bound to matrix or host cell receptors, is translocated posteriorly. It has been hypothesized that these adhesive interactions must ultimately be disengaged for continuous forward movement to occur. TRAP has a canonical rhomboid-cleavage site within its transmembrane domain and mutations were introduced into this sequence to elucidate the function of TRAP cleavage and determine the nature of the responsible protease. Rhomboid cleavage site mutants were defective in TRAP shedding and displayed slow, staccato motility and reduced infectivity. Moreover, they had a more dramatic reduction in infectivity after intradermal inoculation compared to intravenous inoculation, suggesting that robust gliding is critical for dermal exit. The intermediate phenotype of the rhomboid cleavage site mutants suggested residual, albeit inefficient cleavage by another protease. We therefore generated a mutant in which both the rhomboid-cleavage site and the alternate cleavage site were altered. This mutant was non-motile and non-infectious, demonstrating that TRAP removal from the sporozoite surface functions to break adhesive connections between the parasite and extracellular matrix or host cell receptors, which in turn is essential for motility and invasion.

## Author Summary

Malaria infection begins with the bite of an infected mosquito which inoculates sporozoites into the skin. Sporozoites then go to the liver where they invade hepatocytes and replicate, ultimately leading to the blood stage of infection. Sporozoites are motile and actively invade hepatocytes using a unique form of motility called gliding motility. The mechanism by which the parasite moves forward is somewhat similar to a treadmill and the sporozoite protein TRAP, is key to this process. Its extracellular portion binds to host proteins while its intracellular portion binds to the parasite's motor. As the motor moves the protein rearwards, the sporozoite moves forward. It follows that the extracellular adhesive interactions of TRAP must ultimately be disengaged for forward movement to occur. We have generated mutant sporozoites that can only partially disengage these parasite-host adhesive interactions and find that these sporozoites have a halting, constipated movement. Following this, we generated a mutant that cannot disengage these interactions at all and these sporozoites are nonmotile and noninfectious. Lastly we found that a parasite rhomboid protease, ROM4, is on the surface of the sporozoite and thus may be responsible for TRAP cleavage and shedding from the sporozoite surface. Overall, our results demonstrate that robust gliding motility requires the disengagement of adhesive interactions.

## Introduction

Malaria is one of the most important infectious diseases worldwide, causing an estimated 500 million clinical cases and 800,000 deaths annually [1]. *Plasmodium* species, the causative agents of malaria, belong to the phylum Apicomplexa, whose members include other human pathogens such as *Toxoplasma gondii* and *Crytosporidium* species. The Apicomplexans are obligate intracellular parasites and the invasive stages of these protists, called zoites, actively enter host cells using a unique form of locomotion called gliding motility.

Gliding motility is a substrate-dependent form of locomotion that does not involve significant change in cell shape and is powered by a subpellicular actomyosin system linked to the zoite surface through one or more members of the Thrombospondin Related Anonymous Protein (TRAP) family (reviewed in [2], [3]). TRAP family members are type I transmembrane proteins bearing extracellular adhesive domains and a cytoplasmic domain that recruits the glycolytic enzyme aldolase which in turn binds to F-actin and hence connects to myosin A [4], [5]. The forward locomotion of the zoite results from the posterior translocation of TRAP-aldolase-actin assembly. In the rodent malaria parasite, *Plasmodium berghei*, deletion of TRAP or mutations in its cytoplasmic domain that abrogate its interaction with aldolase result in non-motile sporozoites [6], [7].

Generation of nonmotile sporozoites linked gliding motility to host cell invasion, supporting earlier findings in *Toxoplasma* that apicomplexan zoites actively invade host cells [6], [8]. Zoites also require motility to reach their target cell and vary tremendously in the degree to which they are reliant on motility in this regard. *Plasmodium* merozoites, for example, are released in close proximity to their target cell and although they possess all of the motor components and likely use this machinery to invade cells [9], they are not capable of gliding motility in vitro. In contrast, *Plasmodium* sporozoites develop in oocysts on the mosquito midgut wall, far from their ultimate target, the mammalian liver. They must enter mosquito salivary glands, from where they are inoculated into the mammalian dermis, exit the dermis to enter the blood circulation and finally penetrate the sinusoidal barrier of the liver to reach the hepatocytes. In vitro they display a robust gliding phenotype that parallels their need to move longer distances compared to merozoites, ookinetes and zoites of other Apicomplexan genera.

Proteolytic cleavage of surface proteins is a central feature of invasion by apicomplexans (reviewed in [10]). Cleavage occurring in the amino-terminus exposes critical adhesive motifs [11] whereas carboxy-terminal cleavage is thought to disengage adhesive interactions between parasite ligands and host cell receptors (reviewed in [10]). Carboxy-terminal cleavage can occur either extracellularly, close to the plasma membrane, or within the transmembrane domain of the protein, with distinct classes of serine proteases being responsible in each case. In *Plasmodium*, removal of adhesins from merozoites has been studied in some detail. Two of the most abundant merozoite surface proteins, merozoite surface protein 1 (MSP1) and apical membrane protein 1 (AMA1) are removed by a subtilisin-like protease called SUB2 which cleaves its substrates in a juxtamembrane location [12], [13] whereas the invasion ligand EBA-175 is cleaved within its transmembrane domain [14]. Intramembraneous cleavage is accomplished by rhomboid proteases, a family of serine proteases initially described in *Drosophila* that require helical instability in the transmembrane domain and have specific residue requirements in their P1, P4 and P2′ positions [15], [16]. Initial studies demonstrating a role for intramembraneous cleavage of zoite adhesins were carried out in *Toxoplasma* where it was shown that the microneme proteins TgMIC2, TgMIC6 and TgMIC12 were shed from the zoite surface after cleavage within their transmembrane domain [17], [18], [19]. More recently, a conditional knockout of the rhomboid protease TgROM4 demonstrated that this protease plays a critical role in cleavage of TgMIC2 and TgAMA1 with downstream effects on motility, invasion and parasite replication [20], [21]. Importantly, a conserved rhomboid substrate motif is found in all TRAP family members [17] .

To date, the role of cleavage and shedding of surface adhesins in *Plasmodium* sporozoites has not been addressed. However, a previous study has shown that TRAP is cleaved and shed into the supernatant after incubation of sporozoites at 37°C [22]. Moreover, when expressed in heterologous systems, TRAP can be cleaved by a rhomboid protease [18], [23]. Considering that an ortholog of *TgROM4* is found in all malaria parasite genomes and TRAP contains a canonical rhomboid cleavage site in its transmembrane domain, it is plausible that TRAP is shed from the sporozoite surface by the action of a rhomboid. In this study, we generated a series of *TRAP* mutants in the rodent malaria parasite, *P.berghei*, and performed functional and biochemical assays to elucidate the importance of TRAP cleavage and to characterize the nature of the protease responsible for this event.

## Results

### TRAP is proteolytically processed and shed from the sporozoite surface by a serine protease

**Figure 1:**
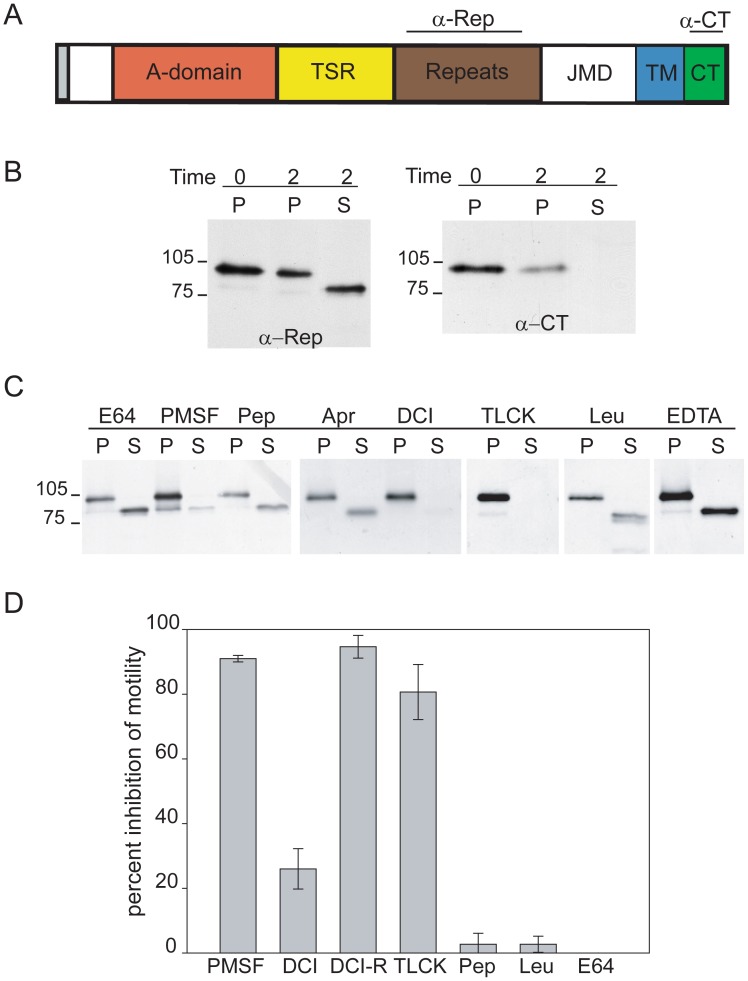
TRAP is proteolytically processed and shed from the sporozoite surface by a serine protease. (A) Primary Structure of TRAP: Shown are the extracellular adhesive domains, namely the A-domain and the type I thrombospondin repeat (TSR), as well as the repeat region, the juxtamembrane region (JMD), the transmembrane domain (TM) and cytoplasmic tail (CT). Anti-TRAP antibodies used in this study recognize either the repeat region (α-Rep) or the cytoplasmic tail of TRAP (α-CT). (B) Pulse-chase metabolic labeling and TRAP immunoprecipitation using anti-repeat or anti-cytoplasmic tail antisera. Salivary gland sporozoites were metabolically labeled and placed on ice for 2 hrs (Time = 0) or chased at 28°C for 2 hrs (Time = 2). Sporozoites were then centrifuged and TRAP was immunoprecipitated from either the pellet (P) or supernatant (S) using antibodies against the repeat region of TRAP (left panel) or antibodies against the cytoplasmic tail (right panel) and analyzed by SDS-PAGE and autoradiography. Supernatants from control sporozoites kept on ice did not contain any TRAP (data not shown). (C) Effect of protease inhibitors on TRAP cleavage. Salivary gland sporozoites were metabolically labeled and chased at 28°C for 2 hrs in the presence of the indicated protease inhibitors. Sporozoites were then centrifuged and TRAP was immunoprecipitated from either the pellet (P) or supernatant (S) using anti-repeat antisera and analyzed by SDS-PAGE and autoradiography. The following inhibitors were used: 10 µM E64, 1 mM PMSF, 1 µM pepstatin (Pep), 0.3 µM aprotinin (Apr), 100 µM 3,4 DCI, 100 µM TLCK, 75 µM leupeptin (Leu), and 5 mM EDTA. (D) Effect of protease inhibitors on gliding motility. Salivary gland sporozoites were pre-incubated with the indicated protease inhibitors and then added to slides in the continued presence of the inhibitor for 1 hr at 37°C. Sporozoite trails were visualized and the number of sporozoites with and without trails was counted. Inhibition of motility was calculated based on the motility of sporozoites pre-treated with media alone. Each inhibitor was tested in triplicate and 50 fields per well were counted. The means ± SD are shown. DCI-R indicates that DCI was replenished every 20 min. All inhibitors were tested in at least two independent experiments however DCI and PMSF were tested in 3 or more independent experiments. A representative experiment is shown.

To analyze TRAP processing, we performed pulse-chase metabolic labeling experiments with sporozoites, and immunoprecipitated TRAP from the sporozoite pellet and supernatant using antibodies specific for the repeat region (α-Rep, Fig. 1A). An ∼83 kD species was associated with the sporozoite pellet whereas TRAP processing led to the release of a ∼76 kD species into the supernatant (Fig. 1B). The recognition of the 76 kD product released into the supernatant by anti-repeat antibodies suggests that the extracellular domain of TRAP is shed, in agreement with previous findings [22]. When antisera recognizing the cytoplasmic tail of TRAP (α-CT, Fig. 1A) was used to immunoprecipitate TRAP, full-length TRAP associated with the sporozoite pellet could be detected but the cleaved portion was not detected in the supernatant, indicating that TRAP is shed without its C-terminal domain (Fig. 1B). These data suggest that TRAP is cleaved either within its transmembrane domain or in the juxtamembrane region.

To determine the nature of the protease responsible for TRAP cleavage, we examined the effect of a variety of protease inhibitors on this process. As shown in Figure 1C, TRAP cleavage was inhibited by a subset of serine proteases inhibitors, namely TLCK, PMSF, and DCI but was not affected by EDTA, cysteine and aspartyl protease inhibitors, or the serine protease inhibitors leupeptin and aprotinin. Overall these data suggest that TRAP is cleaved by a calcium-independent serine protease.

Since TRAP was previously shown to play a critical role in gliding motility [6], we determined if these protease inhibitors also impact on motility. When sporozoites were preincubated with protease inhibitors and then kept in their presence during a gliding motility assay, we found that TLCK and PMSF blocked gliding. DCI, which has a short half-life, had a moderate effect on motility, however, when it was replenished during the assay the inhibition was stronger (Fig. 1D). Protease inhibitors that had no effect on TRAP processing, namely, pepstatin, leupeptin, and E-64, also had no inhibitory effect on motility. Since the same subset of serine protease inhibitors had inhibitory effects on both TRAP cleavage and gliding motility, our data suggest that the removal of TRAP from the sporozoite surface is required for gliding motility.

### Disruption of the rhomboid motif impairs TRAP cleavage

**Figure 2:**
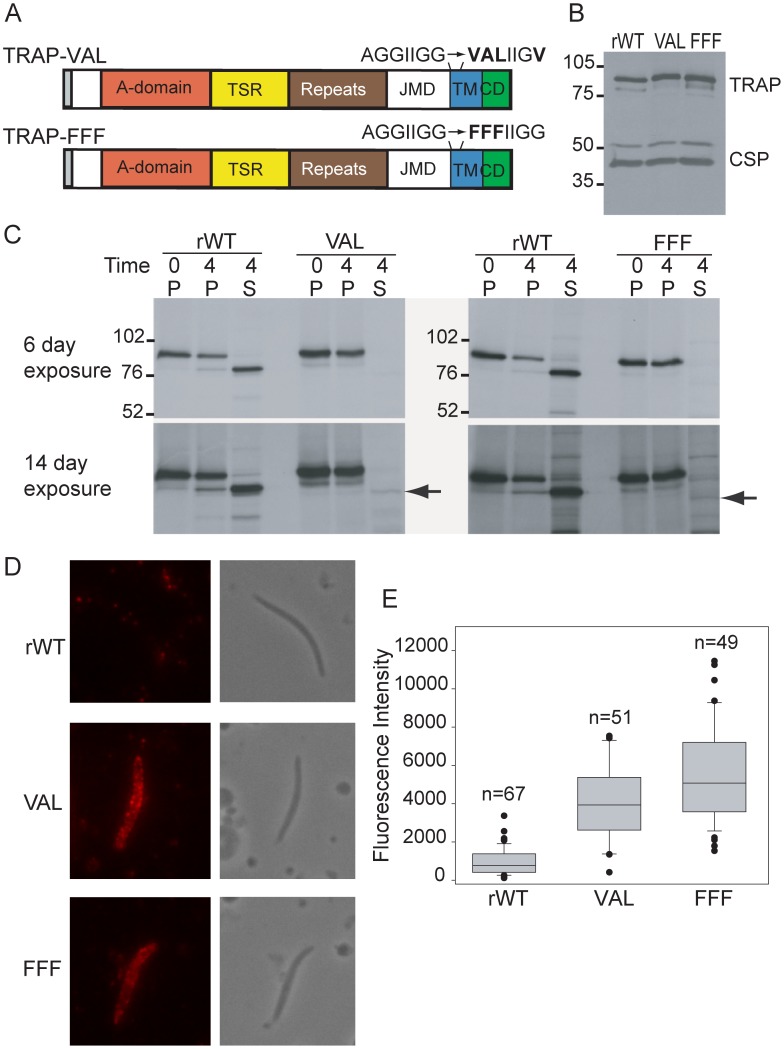
Disruption of the rhomboid motif impairs TRAP cleavage. (A) Primary structure of TRAP expressed in TRAP-VAL and TRAP-FFF mutants, with the point mutations introduced to disrupt the putative rhomboid substrate motif indicated above each transmembrane domain. (B) Western blot analysis of recombinant control TRAP-rWT (rWT) and rhomboid cleavage mutant salivary gland sporozoites TRAP-VAL and TRAP-FFF (VAL and FFF) probed with TRAP anti-repeat antisera. As a loading control the bottom half of the membrane was probed with mAb 3D11 which recognizes the repeat region of CSP. (C) Pulse-chase metabolic labeling of TRAP-rWT and rhomboid cleavage site mutants. Salivary gland sporozoites were metabolically labeled for 1 hr and either placed on ice for 4 hrs (Time = 0) or chased for 4 hrs (Time = 4). Sporozoites were then centrifuged and TRAP was immunoprecipitated from either the pellet (P) or supernatant (S) using anti-repeat antisera and analyzed by SDS-PAGE and autoradiography. Molecular weight markers shown on left of top panel. Top panel: 6 day exposure. Bottom panel: 14 day exposure, arrows show location of processed VAL and FFF mutant TRAP. (D) Immunofluorescence analysis of surface TRAP staining in TRAP-rWT and rhomboid cleavage site mutants. Shown are representative fluorescence and phase contrast images of the TRAP staining pattern after fixation with paraformaldehyde. Microscope and camera settings were identical for all photographs. (E) Box plot of fluorescence intensity of TRAP surface staining in TRAP-rWT and rhomboid cleavage site mutants. Unpermeabilized sporozoites were stained with anti-TRAP repeat antisera and intensity of staining was measured using NIS Elements software. Identical camera and microscope settings were used for all measurements. Boxes contain 50% of the data around its median (black line in box). Whiskers show the range of data within the 10^th^ and 90^th^ percentiles and outliers are shown individually. Results are pooled from 2 to 4 independent experiments. There was a statistically significant difference in staining intensity between TRAP-rWT and TRAP-VAL sporozoites (p<.0001) and between TRAP-rWT and TRAP-FFF sporozoites (p<.0001).

In order to elucidate the function of TRAP cleavage and to better define the nature of the responsible protease, we generated sporozoites expressing mutated forms of TRAP in which point mutations were introduced in the transmembrane domain to disrupt the putative rhomboid substrate motif. We created two rhomboid cleavage site mutants based on previously published studies in *Toxoplasma* and *Plasmodium* in which these mutations led to aberrant or impaired release of the rhomboid protease substrate from the zoite surface [14], [18], [19]: One in which the canonical rhomboid motif AGGIIGG was changed to VALIIGV (TRAP-VAL; Fig. 2A) and another in which it was changed to FFFIIGG (TRAP-FFF; Fig. 2A). Targeting plasmids were designed to replace the endogenous locus via double-cross-over homologous recombination (Fig. S1A). A recombinant control line (TRAP-rWT) was generated using a plasmid containing a wild type copy of the *TRAP* open reading frame. After transfection and cloning, a series of diagnostic PCRs and sequencing was used to verify integration into the correct genomic locus and the presence of the desired mutations (Fig. S1B). TRAP-rWT parasites were similar to wild type *P. berghei* ANKA parasites and were used throughout this study for comparison to mutant lines. Western blot analysis of the rhomboid cleavage site mutants demonstrated that the parasites express normal amounts of TRAP compared to controls (Fig. 2B).

### Mutations in the rhomboid cleavage site impair TRAP processing and led to its accumulation on the sporozoite surface

We performed pulse-chase metabolic labeling experiments to assess TRAP processing in the rhomboid cleavage site mutants and found that cleavage of TRAP was severely impaired in the two mutant lines (Fig. 2C). We then examined the cellular localization of TRAP in the mutant sporozoites by immunofluorescence microscopy. Total overall TRAP staining in permeabilized sporozoites was similar in mutants and controls (data not shown), consistent with the Western blot results. However, when only surface TRAP was stained, a striking difference was observed between mutant and control sporozoites. In wild type sporozoites, there is typically only a small amount of TRAP found on the sporozoite surface, and the staining pattern can be described as a “faint dusting” [24]. In contrast, the majority of rhomboid-cleavage site mutants displayed larger amounts of TRAP on their surface with bright staining along most of their surface (Figs. 2D & 2E), indicative of an absence of shedding. Quantitative measurement of fluorescence intensity of these stained sporozoites was revealed a statistically significant difference between the rhomboid cleavage site mutants and TRAP-rWT sporozoites (p<.0001).

### TRAP processing is critical for salivary gland invasion

Given the impaired TRAP processing and shedding in the rhomboid cleavage site mutants, we set out to analyze the phenotype of these mutants. Since TRAP is not expressed in erythrocytic stages and a previous study in which the *TRAP* gene had been deleted did not show an altered phenotype in these stages [6], we did not expect altered growth of asexual erythrocytic stages or gametocyte production in these mutants and this was indeed the case (data not shown). To study the mosquito stages, *Anopheles stephensi* mosquitoes were allowed to feed on infected mice and sporozoites were isolated from mosquito midguts, hemolymph, and salivary glands for analysis. Sporozoites develop in oocysts on the mosquito midgut wall and reach maximum numbers on day 14 post blood-meal. They are then released into the hemolymph from where they specifically bind to and invade salivary glands, where they reach maximal numbers on day 18 post blood-meal. As shown in Table 1, numbers of oocyst and hemolymph sporozoites were comparable in mosquitoes infected with TRAP-rWT, TRAP-VAL or TRAP-FFF parasites, indicating that the introduced mutations had no effect on sporozoite development in the oocyst or their release into the hemolymph. However, when salivary gland sporozoite populations were examined, both TRAP-VAL and TRAP-FFF clones had five times fewer salivary gland sporozoites compared to the TRAP-rWT clones, suggesting a defect in invasion (Table 1).

**Table 1:** Sporozoite numbers and localization in mosquitoes infected with control and mutant parasites.

Parasite Line	Midgut Sporozoites*	Hemolymph Sporozoites*	Salivary Gland Sporozoites*	Percent Inside#
**TRAP-rWT**	36,500±200	6,500±1000	10,400±200	82.5%
**TRAP-VAL**	36,400±5900	7,500±3700	2,200±400	77%
**TRAP-FFF**	41,800±3000	6,500±2900	1,400±800	73%
**TRAP-JMD**	49,200±6300	6,000±2500	13,100±1700	77%
**TRAP-DMut**	50,800±2700	7,100±2700	500±200	15%

*Mosquitoes were infected with the indicated parasite clones and midguts, hemolymph and salivary glands were harvested from 15 mosquitoes at days 14, 16 and 18 post-infective blood meal respectively, pooled and sporozoites were counted. Shown are the means of three independent experiments ± SD.

# Sporozoites in the supernatant and pellet of trypsin-treated salivary glands were counted and the percentage inside was calculated. There were 15 mosquitoes per group. This experiment was performed twice with similar results.

### Impaired TRAP processing leads to aberrant gliding motility

**Figure 3:**
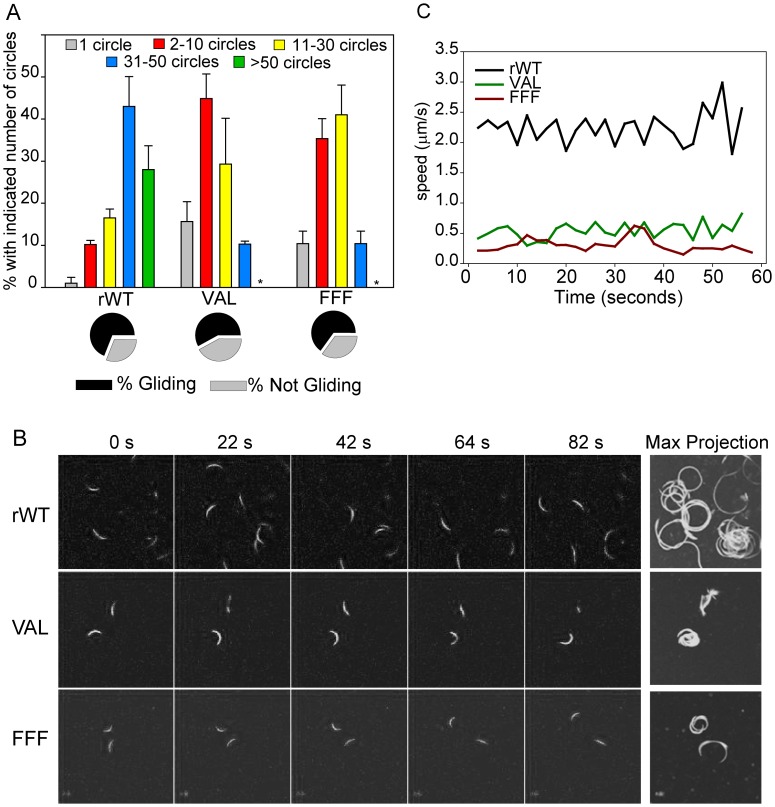
Impaired TRAP processing leads to aberrant gliding motility. (A) Gliding motility of rhomboid cleavage site mutants. Salivary gland sporozoites were incubated on slides for 1 hr and trails were visualized and counted. The percentage of sporozoites with and without trails is shown in the pie charts. For those sporozoites associated with trails, the number of circles produced by each sporozoite was counted and shown is their distribution for each parasite line. Asterisks indicate that none of the TRAP-VAL and TRAP-FFF mutants were associated with over 50 circles. Over 100 sporozoites per well were counted and shown are the means of triplicate wells ± SD. (B) Live imaging of gliding motility of rhomboid cleavage site mutant sporozoites. Sporozoites were observed and recorded using a Leica laser scanning confocal microscope. Time lapse images of sporozoites gliding on glass bottom dishes are shown with the maximum intensity projection on the right. (C) For each parasite line the average speed of ten sporozoites was determined for 60 s. All experiments were performed at least twice and a representative experiment is shown.

To determine the role of TRAP cleavage and shedding on motility, we performed gliding motility assays with the rhomboid cleavage site mutant sporozoites. Initial experiments assayed motility by staining and counting trails left by gliding sporozoites. Staining trails for CSP or TRAP showed that mutants were capable of gliding and leaving trails in their wake (data not shown and Fig. S3). When these trails were counted, we found only a small decrease in the percentage of mutants that were non-motile (Fig. 3A, pie charts). However, when comparing the number of trails produced by each parasite line, rhomboid-cleavage site mutants produced fewer trails: Whereas over 40% of control sporozoites produced 31–50 circles and 25% produced greater than 50 circles, only 10% of mutant sporozoites produced 31–50 circles and none produced more than 50 circles (Fig. 3A bar graph).

To further analyze gliding motility of mutant sporozoites we performed live imaging studies. Like control sporozoites, the rhomboid cleavage site mutants moved in circles, however, they moved at a slower rate and frequently appeared stuck, attempting to move forward but unable to (Videos S1, S2 and S3). The mutants also displayed patterns of non-productive motility, such as bending, flexing, waving, and pendulum-like movements, which consists of moving one-third of a circle and then returning to the starting position (Fig. 3B, Videos S1, S2 and S3). When we calculated their speed, control sporozoites glided with an average speed of ∼2 µm/s, whereas mutant sporozoites glided at a rate of ∼0.5 µm/s (Fig. 3C). Overall these data indicate that the rhomboid cleavage site mutant sporozoites are impaired in motility, traveling shorter distances and at a reduced speed.

### Impaired TRAP processing leads to impaired host cell invasion

**Figure 4:**
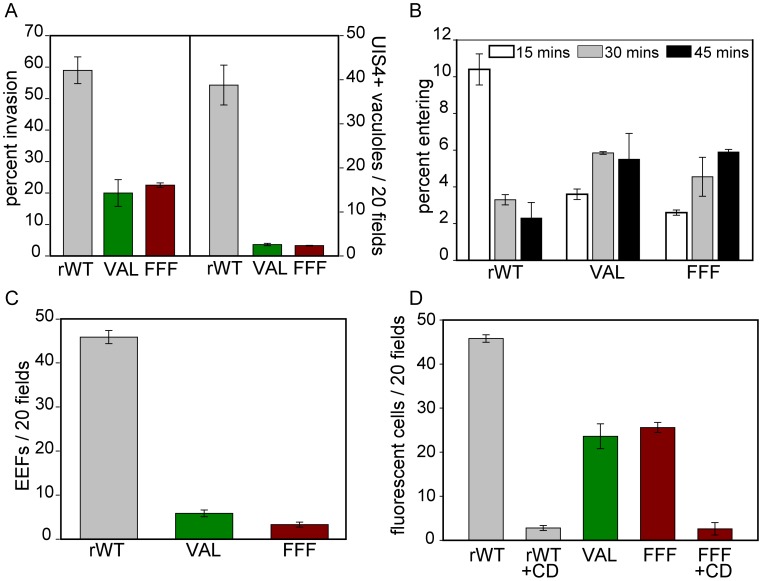
Impaired TRAP processing of rhomboid cleavage site mutants leads to impaired host cell invasion. (A) In vitro invasion. Salivary gland sporozoites were incubated with Hepa 1–6 cells and fixed after 1 hr (data on left) or 6 hrs (data on right). Cells fixed after 1 hr were stained with a double staining assay that distinguishes between extracellular and intracellular sporozoites and the percent of total sporozoites that were intracellular was determined (left axis). Cells fixed after 6 hrs were stained with UIS4 antisera to determine the number of sporozoites that had entered in a vacuole (right axis). For both experiments at least 50 fields per well were counted and shown are the means ± SD of duplicate wells. (B) Kinetics of entry into hepatocytes. Salivary gland sporozoites were incubated with Hepa 1–6 cells for 15, 30 and 45 mins before being washed, fixed and stained with a double-staining assay that distinguishes extracellular and intracellular sporozoites. Shown is the percent of total sporozoites that were in the process of entering host cells, i.e. partially inside and partially outside. 50 fields per coverslip were counted and the means of duplicates ±SD are shown. (C) EEF development. Salivary gland sporozoites were added to Hepa 1,6 cells and incubated for 48 hrs at which time they were fixed and stained. The number of EEFs in 50 fields per coverslip were counted and shown are the means ± SD of duplicate wells. (D) Cell traversal. Salivary gland sporozoites were incubated with Hepa 1,6 cells for 1 hr, in the presence of the nucleic acid dye TOTO-1. Controls were pre-incubated and kept in the presence of cytochalasin D (CD), which inhibits motility. The number of TOTO-1 positive cells in 50 fields was counted and the means ±SD of duplicate wells are shown. All experiments were performed at least twice and representative experiments are shown.

Since gliding motility is required for host cell invasion [6], [8], we examined the infectivity of the rhomboid cleavage site mutants in a number of in vitro assays. First we determined the invasion rate by counting the number of intracellular and extracellular sporozoites after their incubation with the hepatocyte cell line, Hepa1-6. The mutant sporozoites displayed a marked decrease in invasion with 20% of mutant sporozoites compared to 60% of control sporozoites being found intracellularly (Fig. 4A, left axis). One limitation of this assay, however, is that it fails to distinguish between sporozoites that have productively invaded the cells, i.e. with the formation of a parasitophorous vacuole (PV) versus sporozoites that are only migrating through, a process that is distinct from productive invasion and results in wounding of the traversed cell [25]. To address this issue, we performed invasion assays and stained intracellular sporozoites with antisera to UIS4, a protein that is localized to the PV membrane [26]. Only a small proportion of the intracellular TRAP-VAL and TRAP-FFF sporozoites stain with UIS4 (Fig. 4A, right axis), indicating that the majority of the mutants are not able to productively invade hepatocytes. This was confirmed when we tested the ability of the rhomboid cleavage site mutants to develop into exoerythrocytic stages (EEFs) in vitro. There was an approximately 10-fold reduction in the number of EEFs produced by the mutant sporozoites compared to controls, correlating with the small percentage of mutant sporozoites that productively invade hepatocytes (Figs. 4 A&C). However, the few EEFs that are formed are similar in size to control EEFs (data not shown), indicating that TRAP cleavage does not play a role in EEF development.

Since the gliding motility studies demonstrated that the rhomboid cleavage site mutants moved more slowly, we also examined the kinetics with which these mutants invade hepatocytes. The number of sporozoites in the process of entering hepatocytes after 15, 30, and 45 minutes was quantified by counting sporozoites that were half in and half out. The highest percentage of control TRAP-rWT sporozoites in the process of entering was seen at 15 minutes after their addition to cells and this number decreased over time. Conversely, the percentage of TRAP-VAL and TRAP-FFF sporozoites entering hepatocytes was lowest at 15 minutes and increased slightly at later time points (Fig. 4B). As stated above, this inside/outside assay cannot distinguish sporozoites that are productively invading versus those that are migrating through. Nonetheless, these data indicate that cell entry for either of these processes, is slower for the rhomboid cleavage site mutants, a finding that is consistent with their gliding phenotype.

### Impaired TRAP processing leads to decreased infectivity in the mammalian host

Infectivity of the rhomboid-cleavage site mutants was evaluated in mice after both intravenous (i.v.) and intradermal (i.d.) inoculation. We first determined the time to detection of blood stage parasites (prepatent period) after i.v. inoculation into Swiss Webster mice and found that the rhomboid cleavage site mutants had a delay of one to two days in the prepatent period compared to controls (Table 2). Since each day delay is correlated with a 10-fold decrease in infectivity [27], our results indicate that the mutant sporozoites were ∼10 to 100-fold less infective than control sporozoites in these mice. We also examined infectivity of these mutants in C57BL/6 mice, which are highly susceptible to *P. berghei* infection, and found a one day delay in the prepatent period compared to controls (Table 2). When we tested the infectivity of the rhomboid cleavage site mutants after i.d. inoculation, the decrease in infectivity was more pronounced. In all of the Swiss Webster mice and the majority of the C57BL/6 mice, blood stage parasites were not observed after i.d. inoculation of the mutant sporozoites, with monitoring up to 21 days post injection (Table 2). The one C57BL/6 mouse in each group that did become positive for blood stage parasites had a significant delay in patency. Since sporozoites are inoculated by mosquitoes into the dermis of the mammalian host [28], [29] and migration through cells and tissues is critical for sporozoite exit from the dermis [30], [31], [32], this dramatic decrease in infectivity after i.d. inoculation suggests that robust/fast gliding motility is particularly important for dermal exit. To further evaluate the ability of the rhomboid cleavage site mutants to migrate through cells, we performed an in vitro assay in which sporozoites are added to cells in the presence of a fluorescent cell-impermeant dye. Wounded cells take up the dye and can then be counted [25]. As shown in Figure 4D, mutant sporozoites exhibited a reduction in cell traversal activity compared to control sporozoites.

**Table 2:** In vivo infectivity of TRAP mutant sporozoites as determined by prepatent period.

	Mouse Strain	Parasite Line	Route of Inoculation	Number Sporozoites Injected	# Mice Positive	Prepatent Period (days)
					# Mice Injected	
Expt. 1	Swiss Webster	rWT	IV	500	4/5	4.0
			IV	5000	5/5	3.0
		VAL	IV	500	3/5	5.6
			IV	5000	5/5	4.6
		FFF	IV	500	3/5	6.0
			IV	5000	4/5	5.0
Expt. 2	C57BL/6	rWT	IV	100	5/5	4.2
			IV	1000	5/5	3.0
			IV	10,000	5/5	3.0
		VAL	IV	100	5/5	5.0
			IV	1000	5/5	4.0
			IV	10,000	5/5	3.5
		FFF	IV	100	5/5	5.0
			IV	1000	5/5	4.0
			IV	10,000	5/5	4.0
Expt. 3	Swiss Webster	rWT	ID	5000	4/5	3.2
		VAL	ID	5000	0/5	-
		FFF	ID	5000	0/5	-
Expt. 4	C57/BL6	rWT	ID	5000	5/5	3.0
		VAL	ID	5000	0/5	-
			ID	25,000	1/5	6.0
		FFF	ID	5000	1/5	7.0
			ID	25,000	0/5	-
Expt. 5	Swiss Webster	JMD	IV	10,000	5/5	3.4
		DMut	IV	10,000	0/5	-
			IV	25,000	0/3	-
Expt. 6	C57/BL6	JMD	IV	1000	5/5	3.0
		DMut	IV	10,000	0/5	-
			IV	27,000	0/2	-

### Creation of an uncleavable TRAP by deletion of the juxtamembrane portion and mutation of the rhomboid cleavage site

Although the point mutations introduced within the putative rhomboid cleavage site of TRAP resulted in impaired cleavage, parasite motility and invasion, these activities were not completely abolished. It is conceivable that a small amount of TRAP is still cleaved, at an alternate site, allowing for the slow, halting movement we observed. Indeed, overexposure of the film from the pulse-chase experiment shown in Figure 2 indicated residual processing of TRAP (Fig. 2C, black arrow). Although we cannot isolate sufficient amounts of protein to map either the canonical or the alternate cleavage sites, the size of the shed form of TRAP-VAL and TRAP-FFF was not significantly smaller than the shed form of TRAP-rWT, suggesting that mutant TRAP was being cleaved close by, possibly in a juxtamembrane location.

**Figure 5:**
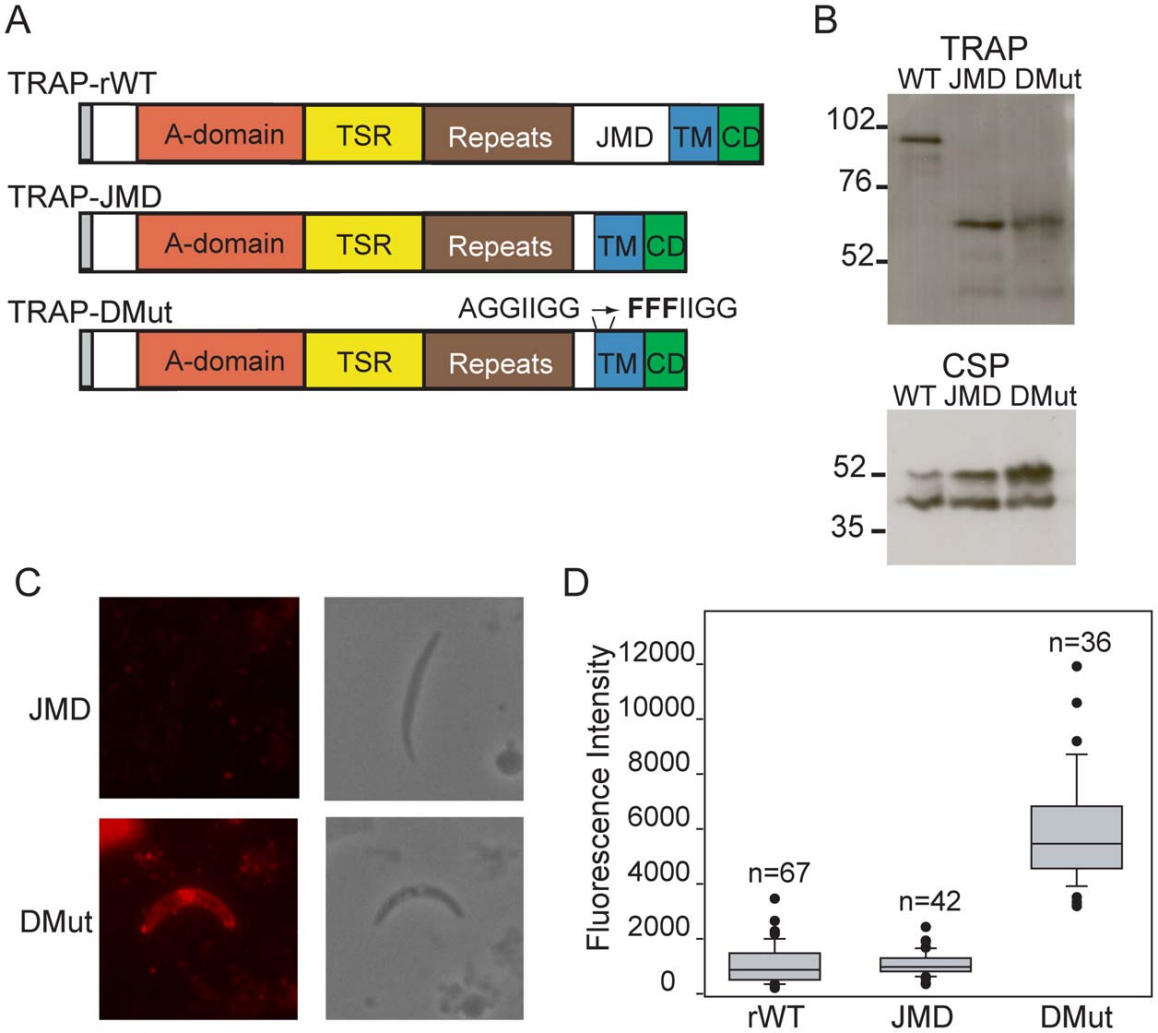
Inefficient cleavage of TRAP may be due to alternate cleavage in the juxtamembrane region. (A) Primary structure of TRAP expressed in TRAP-JMD and TRAP-DMut sporozoites. TRAP-JMD has 140 amino acids of the juxtamembrane region deleted and an intact wildtype rhomboid substrate motif. The double mutant, TRAP-DMut has both 140 amino acids of the juxtamembrane region deleted and an altered rhomboid substrate motif. The point mutations introduced to disrupt the rhomboid substrate motif are shown. (B) Western blot analysis of TRAP-rWT, TRAP-JMD and TRAP-DMut sporozoites. Sporozoite lysates from midgut sporozoites were separated by SDS-PAGE, transferred to a PVDF membrane and probed with TRAP anti-repeat antisera. As a loading control, the membrane was stripped and probed with mAb 3D11, which recognizes the repeat region of CSP. (C) Immunofluorescence analysis of surface TRAP staining in TRAP-JMD and TRAP-DMut salivary gland sporozoites. Shown are representative fluorescence and phase contrast images of the TRAP staining pattern after fixation with paraformaldehyde. Microscope and camera settings were identical for all photographs. (D) Box plot of fluorescence intensity of TRAP surface staining in TRAP-JMD and TRAP-DMut salivary gland sporozoites with data from TRAP-rWT included for comparison. Unpermeabilized sporozoites were stained with anti-TRAP repeat antisera and intensity of staining was measured using NIS Elements software. Identical camera and microscope settings were used for all measurements. Boxes contain 50% of the data around its median (black line in box). Whiskers show the range of data within the 10^th^ and 90^th^ percentiles and outliers are shown individually. Results are pooled from 2 to 4 independent experiments. There was a statistically significant difference in staining intensity between TRAP-rWT and TRAP-DMut sporozoites (p<.0001) and between TRAP-JMD and TRAP-DMut sporozoites (p<.0001). There was no statistically significant difference in staining intensity between TRAP-rWT and TRAP-JMD sporozoites or between TRAP-DMut and TRAP-FFF sporozoites.

In *Plasmodium* merozoites, subtilisin-like protease PfSUB2 has been implicated in shedding of adhesins at a juxtamembrane position [13]. The known substrates for *Plasmodium* subtilisins harbor no obvious sequence similarity. Instead, it has been proposed that subtilisin-substrate recognition involves stretches of conformationally unrestrained peptides around the target peptide bond such that the degree of “disorder” rather than a primary amino acid sequence serves as a determinant for cleavage [12]. The juxtamembrane region of *P. berghei* TRAP, which starts downstream of the proline based repeats, consists of approximately 160 amino acids (Fig. S2) and is predicted to be disordered and with low complexity. To test whether this region can serve as a substrate for cleavage, we generated two additional TRAP mutants. TRAP-DMut corresponds to a double mutant (Fig. 5A and Fig. S2) in which 140 amino acids of the juxtamembrane region were deleted and the rhomboid substrate motif, AGGIIGG was changed to FFFIIGG. While no function has been ascribed to the juxtamembrane region of TRAP, it is possible that deletion of this large portion of the protein may affect protein stability, expression and function. Therefore, we also generated a control mutant line, TRAP-JMD, in which the same 140 amino acids of the juxtamembrane domain were deleted but the rhomboid substrate motif was maintained (Fig. 5A and Fig. S2). Any defects associated solely with the deletion of the juxtamembrane region could be analyzed with this mutant. Similar to the generation of the rhomboid cleavage site mutants, targeting plasmids containing *TRAP-JMD* and *TRAP-DMut* genes were constructed to replace the endogenous *TRAP* locus and a series of diagnostic PCRs and sequencing was used to verify integration into the *TRAP* locus and the presence of the desired mutations in the cloned transfectants (Fig. S1 and data not shown).

Both mutants expressed normal amounts of TRAP by Western blot, however, its molecular weight was significantly lower due to deletion of the juxtamembrane region (Fig. 5B). TRAP localization in permeabilized TRAP-DMut and TRAP-JMD sporozoites was analyzed by immunofluorescence and was found to be similar to previous controls (data not shown). In contrast, surface staining of TRAP gave strikingly different results for the two mutants. Whereas TRAP-JMD sporozoites were similar to controls, the majority of TRAP-DMut sporozoites expressed large amounts of TRAP on their surface similar to the rhomboid cleavage site mutants (Figs. 5C & 5D). This result suggests that removal of TRAP-DMut from the sporozoite surface is considerably inhibited. Unfortunately, a more quantitative assessment of TRAP cleavage by pulse-chase metabolic labeling was technically not possible due to the limited number of TRAP-DMut salivary gland sporozoites (see below).

Sporozoite development in mosquitoes of TRAP-JMD and TRAP-DMut parasites was similar to wild type parasites, with normal numbers of midgut and hemolymph sporozoites (Table 1). Furthermore, TRAP-JMD mutants had normal numbers of salivary gland sporozoites, indicating that infectivity in the vector was not altered by deletion of the juxtamembrane region of TRAP. In contrast, there were very low numbers of salivary gland TRAP-DMut sporozoites, approximately 30-fold lower than controls and 4-fold lower than TRAP-VAL and TRAP-FFF mutants (Table 1). To examine the phenotype more in depth, we determined the percentage of salivary gland sporozoites that were inside the glands for each mutant. Salivary glands were incubated with trypsin after which sporozoites remaining with the glands and those released into the supernatant were counted. As shown in Table 1, TRAP-JMD parasites and the rhomboid cleavage site mutants were not significantly different from controls, with approximately 77% of salivary gland associated sporozoites found inside the glands. In contrast, there was a dramatic decrease in the proportion of TRAP-DMut sporozoites inside the salivary glands with only 15% found inside (Table 1). Taken together, these data indicate that TRAP-DMut sporozoites are severely impaired in their ability to invade salivary glands.

### Sporozoites expressing an uncleavable TRAP are non-motile and non-infectious

**Figure 6:**
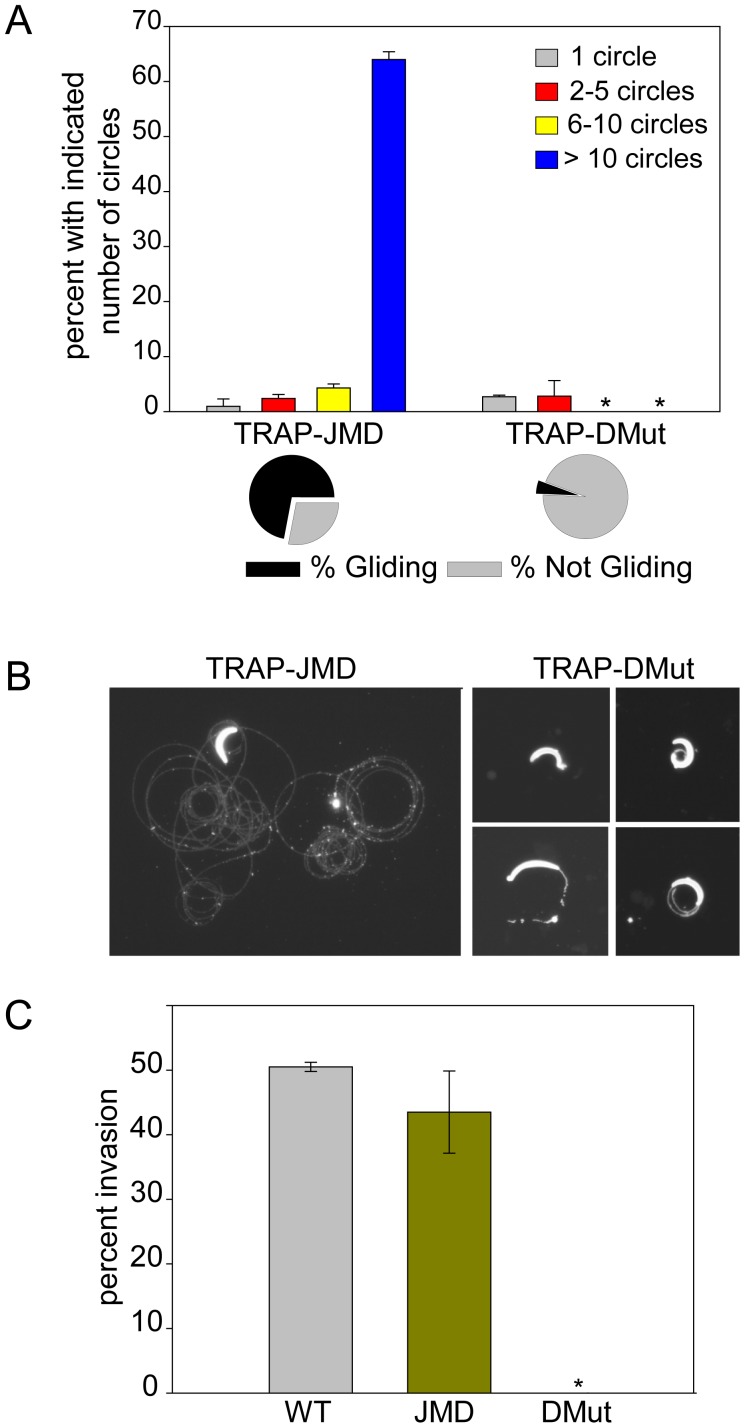
TRAP-DMut sporozoites are severely impaired in gliding motility and host cell invasion. (A) Gliding motility of TRAP-JMD and TRAP-DMut sporozoites. Salivary gland sporozoites were incubated on slides for 1 hr and trails were visualized and counted. The percentage of sporozoites with and without trails is shown in the pie charts. For those sporozoites associated with trails, the number of circles produced by each sporozoite was counted and shown is their distribution for each parasite line. Over 100 sporozoites per well were counted and shown are the means of triplicate wells ± SD. (B) Representative images of the types of trails produced by each mutant. (C) Hepatocyte invasion. Salivary gland sporozoites were incubated with Hepa 1–6 cells for 1 hr, fixed and stained with a double staining assay that distinguishes extracellular and intracellular sporozoites. Percent invasion was determined and shown are the means ± SD of duplicate wells. All experiments were performed at least twice and shown is a representative experiment.

We performed a number of functional assays to determine the phenotype of TRAP-DMut and TRAP-JMD sporozoites in the mammalian host. In gliding motility assays the majority of TRAP-JMD sporozoites were motile and produced greater than 10 circles, similar to controls (Fig. 6 A&B; controls shown in Fig. 3A). In contrast, the majority of TRAP-DMut sporozoites were completely non-motile and the small number that moved produced only 1 to 2 circles (Fig. 6 A&B).

In vitro infectivity assays revealed that TRAP-JMD sporozoites were able to invade hepatocytes with rates similar to those observed with TRAP-rWT sporozoites whereas TRAP-DMut sporozoites did not invade hepatocytes at all (Fig. 6C). When we examined the infectivity of these mutants in mice after i.v. inoculation, we found that TRAP-JMD mutants had similar infectivity to controls in both Swiss Webster and C57Bl/6 mice whereas i.v. inoculation of high doses of TRAP-DMut sporozoites never resulted in a blood stage infection in either mouse strain (Table 2). This complete lack of infectivity of TRAP-DMut sporozoites correlates the absence of gliding and lack of shedding of TRAP from the zoite surface.

### PbROM4 is expressed both in schizonts and sporozoites

**Figure 7:**
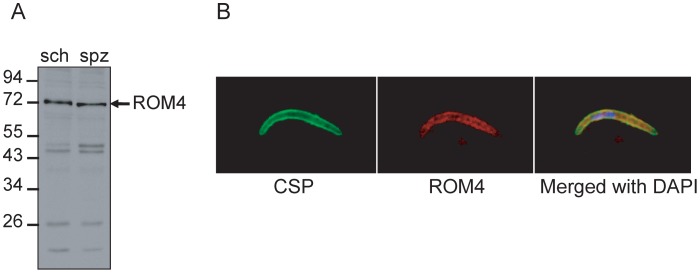
ROM4 is expressed on the sporozoite surface and antibodies against the extracellular tail of ROM4 inhibit hepatocyte invasion. (A) Western blot analysis of *P. berghei* erythrocytic stage schizont lysate (sch) and salivary gland sporozoites (spz) probed with anti-PfROM4 C-terminal IgG. Molecular weight markers are in kDa. (B) Immunofluorescence of *P. berghei* salivary gland sporozoites fixed with cold methanol and stained with anti-CSP antibodies and anti-PfROM4 C-terminal IgG.

The phenotype of the rhomboid cleavage site mutants suggests that a rhomboid protease is primarily responsible for removal of TRAP from the sporozoite surface. The identity of this rhomboid protease remains unknown, however, the transcriptome database shows that two rhomboid proteases are highly expressed in sporozoite stages, namely ROM1 and ROM4 [33]. Previous investigators have demonstrated that ROM1 does not cleave TRAP [34] and that ROM4 cannot be conventionally deleted because it is vital in the blood stages [14]. We therefore made antisera specific for ROM4 to determine whether the protein was expressed in the sporozoite stage. Polyclonal antisera to the exposed C-terminal portion of *P. falciparum* ROM4 cross-reacted with *P. berghei* ROM4 by Western blot, recognizing a ∼69 kD species which corresponds to the predicted size of PbROM4 (Fig. 7A). The specificity of this antiserum was confirmed using PbROM4 conditional knockout parasites (Fig. S4). Furthermore, immunofluorescence experiments showed that PbROM4 was abundantly expressed in sporozoites and co-localized with CSP found on the sporozoite surface (Fig. 7B). These data demonstrate that ROM4 is expressed during the sporozoite stage and future experiments should determine whether it is responsible for TRAP cleavage and shedding from the sporozoite surface.

## Discussion

TRAP provides a link between the extracellular surface and the motor of the parasite, thus allowing the force generated by the motor to be transmitted to the sporozoite surface. This motile force results in posterior translocation of TRAP and the current model predicts that shedding of TRAP would be essential to disengage interactions between the extracellular matrix or host cell receptors and the motor assembly [2], [3]. Here we demonstrate that removal of TRAP from the sporozoite surface is critical for sporozoite motility which in turn is crucial for exit from the inoculation site in the mammalian host and cell invasion in both mosquito and mammalian hosts. Our data suggest that removal of TRAP is accomplished by a rhomboid protease and that when canonical rhomboid activity is prevented, either another protease can modestly compensate for its activity or the rhomboid protease can cleave, albeit less efficiently, at a juxtamembranous site

The TRAP cleavage site mutants that we generated exhibit two distinct phenotypes: The rhomboid cleavage site mutants, TRAP-VAL and TRAP-FFF, displayed an intermediate gliding/infectivity phenotype and TRAP-DMut exhibited an almost complete abrogation of motility and infectivity. Our data suggest that the intermediate gliding phenotype of the rhomboid cleavage site mutants is due to a defect in TRAP shedding from the sporozoite surface. Indeed, both pulse-chase metabolic labeling experiments demonstrate a dramatic decrease in TRAP cleavage and immunofluorescence studies indicate an accumulation of TRAP on the surface of the mutant sporozoites. Furthermore, live imaging studies showed that rhomboid cleavage site mutants could move the length of a sporozoite but then appeared stuck at their posterior end, eventually disengaging and moving forward another sporozoite length only to become stuck again. This ‘constipated’ movement paralleled the inefficient removal of TRAP from their surface. Nonetheless, both the partial phenotype of these mutants and the small amount of cleaved TRAP observed in pulse-chase metabolic labeling experiments suggested that TRAP could be removed from these parasites, albeit slowly and inefficiently. We hypothesized that inefficient TRAP cleavage was being performed by another protease and focused on the subtilisin-like proteases because of their role as sheddases in the erythrocytic stage of the parasite [13]. The genomes of rodent and human *Plasmodium* parasites contain three subtilisin-like protease genes, *sub1*, *sub2* and *sub3*, all of which are transcribed in sporozoites [33]. Since SUB2 removes adhesins from the *Plasmodium* merozoite surface by juxtamembranous cleavage [13], we indirectly tested whether shedding of mutant TRAP was being performed by a subtilisin-like protease by generating a mutant in which both the juxtamembrane domain was deleted and the rhomboid cleavage site was altered (TRAP-DMut). The low numbers of salivary gland sporozoites produced hampered a direct quantitative assessment of the mutations on TRAP cleavage. Nonetheless, immunofluorescence assays indicated that TRAP-DMut sporozoites accumulated TRAP on their surface and functional assays demonstrated that this mutant was non-motile and not infectious in the mammalian host. Since TRAP-JMD sporozoites, in which the putative subtilisin cleavage site was deleted but the rhomboid cleavage site was left intact, had a phenotype similar to wild type sporozoites, the phenotype of TRAP-DMut sporozoites cannot be attributed to the large deletion in the juxtamembrane region. Overall, these data support the hypothesis that disengagement of adhesive interactions through removal of TRAP from the zoite surface is essential for gliding motility. These findings logically complement a recent study in which sporozoite gliding motility was studied by reflection interference contrast microscopy and found to consist of a series of adhesion de-adhesion events in which TRAP plays a critical role [35]. Taken together, our study and the Munter study [35] highlight the delicate balance between adhesion and de-adhesion that must be achieved for fast and effective gliding motility.

The impairment of TRAP removal from the sporozoite surface and its effect on gliding motility had significant downstream effects on target cell invasion in both the mosquito and mammalian hosts. Furthermore, the degree to which motility was affected correlated with the impairment in infectivity. Less expected and equally important, the intermediate gliding phenotype of the rhomboid cleavage site mutants highlights the critical role of fast (and robust) gliding motility for exit from the dermal inoculation site. In comparison to zoites of other *Plasmodium* life cycle stages and other Apicomplexan genera, sporozoites move longer distances and display a more pronounced gliding phenotype that fuels their need to get from the dermis, where they are deposited by an infected mosquito, to the liver. The rhomboid cleavage-site mutants whose motility was slower and appeared to get stuck due to their inability to disengage adhesive interactions, were significantly less infectious after i.d. inoculation compared to i.v. inoculation, demonstrating the importance of robust gliding for tissue migration.

Both rhomboid cleavage site and TRAP-DMut sporozoites accumulated large amounts of TRAP on their surface in contrast to wild type sporozoites which shed most of their surface TRAP. This is similar to the accumulation of TgMIC2 on the *Toxoplasma* tachyzoite surface following depletion of *TgROM4* [20]. It has been postulated that during motility, adhesins are translocated along the zoite surface and removed at the posterior end of the zoite. However if TRAP were only removed from the posterior end of the sporozoite, we would expect to see accumulation at this location. In contrast, we observed large amounts of TRAP all over the zoite surface. In addition, PbROM4, the protease that may be responsible for TRAP shedding, decorates the entire sporozoite surface and is not concentrated at the posterior pole. Our hypothesis is that TRAP removal occurs in a stochastic manner as it is translocated posteriorly and that this pattern of removal results in the smooth gliding pattern observed with wild type sporozoites. In contrast, the rhomboid cleavage site mutants have a slow ‘constipated’ movement and appear to be stuck at their posterior end. These data suggest that the alternate protease responsible for TRAP removal in these mutants may be located posteriorly, resulting in a back-log of adhesin removal and in the observed gliding phenotype. It is not yet known how rhomboid proteases are regulated, however, in the case of *Drosophila* ROM1, there is no evidence of regulation and the rhomboid is active wherever it is present [36]. Thus it is possible that rhomboids are regulated by their spatial localization and perhaps the localization of PbROM4 along the sporozoite surface allows it to act in a constitutive manner much like *Drosophila* ROM1. This is supported by studies with *Toxoplasma* which found that TgROM4 is distributed along the entire parasite surface and disruption of this gene leads to decreased MIC2 processing and accumulation of MIC2 along the tachyzoite surface [17], [19], [20]. Thus in both *Plasmodium* and *Toxoplasma*, ROM4 may function to remove adhesins as they translocate to the rear of the parasite, enabling smooth forward gliding.

Our data suggest that ROM4 may be the protease that is responsible for TRAP shedding from the sporozoite surface. It is expressed in sporozoites and its localization along the sporozoite surface correlates with the accumulation of TRAP-VAL and TRAP-FFF along the entire surface of the rhomboid cleavage site mutants. There are eight rhomboid-like genes in the *P. falciparum* genome and they are numbered to reflect their homology with their counterparts in *T. gondii*. Of these, only PfROMs 1, 3, 4 and 6 are clear orthologs of the *Toxoplasma* ROMs [17]. PfROM1 and PfROM4 are highly expressed in sporozoites [33], making them the most likely candidates for TRAP cleavage. A previous study examining the enzymatic properties of the *Plasmodium* rhomboids in a heterologous mammalian cell system found that co-expression of TRAP with PfROM4 resulted in its release from the cell surface whereas co-expression of TRAP with PfROM1 did not [23]. Recently, *ROM1* was disrupted in *P. berghei* and TRAP processing was unaffected in these sporozoites [34]. These data, together with the data from our study suggest that PbROM4 is the primary candidate for TRAP removal from the sporozoite surface. Nonetheless, definitive proof that PbROM4 is the TRAP sheddase awaits the establishment of experimental conditions to conditionally deplete *PbROM4* in the mosquito stages since the gene cannot be disrupted in intra-erythrocytic stages [14]. Overall these findings reinforce the notion that targeting serine proteases such as the rhomboids and subtilisins, may constitute novel chemotherapeutic targets for malaria. While it has been demonstrated that rhomboid proteases play a role in the erythocytic stage of the parasite life cycle [14], the findings presented here implicate a role for rhomboid proteases, and potentially subtilisins during the pre-erythrocytic stages of malaria. While the erythrocytic stages of the parasite life cycle are responsible for the clinical manifestations of the disease, the sporozoite stage is responsible for establishing infection in the mammalian host. Hence, targeting the rhomboids would not only provide a mechanism for treating malaria, but also for prevention of disease by inhibiting sporozoite infectivity. Although rhomboids are found throughout all kingdoms of life, there is a significant level of diversity among the different classes of rhomboids with potential differences in structure, function and substrate recognition. Perhaps these differences can be exploited to generate specific rhomboid inhibitors that can lead to a new class of anti-malarial drugs.

## Materials and Methods

### Ethics statement

All animal work was conducted in accordance with the recommendations in the Guide for the Care and Use of Laboratory Animals of the National Institutes of Health. The protocol was approved by the NYU School of Medicine Institutional Animal Care and Use Committee (protocol #080413) and by the Johns Hopkins University Animal Care and Use Committee (protocol #M011H467), which is fully accredited by the Association for Assessment and Accreditation of Laboratory Animal Care. All efforts were made to minimize suffering.

### Construction of targeting plasmids for generation of *TRAP* mutant parasites

Primer sequences used in the generation and verification of mutants are listed in Table S1. Transfection plasmids designed to replace the endogenous locus with mutant or wild type *TRAP* were generated using pDEF-hDHFR (www.malaria.mr4.org) containing the human dihydrofolate reductase (hDHFR) selection cassette (Fig. S1). A 576 bp fragment of 5′UTR located 1000 bp upstream of *TRAP* was amplified by PCR using template genomic DNA from *P.berghei* ANKA parasites, with the primer pair, PbTRAP5′UTR-FWD and PbTRAP5′UTR-REV and this was cloned into pDEF-hDHFR upstream of the selection cassette. A second fragment, consisting of 1062 bp of 5′UTR directly upstream of the *TRAP* gene, 1821 bp of *TRAP* open reading frame, and 2057 bp of *TRAP* 3′UTR was amplified by PCR using the primer pairs PbTRAPFWD and PbTRAPREV and Pfu polymerase (Stratagene). This was cloned into pDEF-hDHFR downstream of the selection cassette to generate the plasmid pTRAP, which was then used to generate the *TRAP* mutants using the Quick Change Mutagenesis kit (Stratagene). Generation of each of the rhomboid cleavage site mutants required 2 steps. Primers MUT-TRAP-VAL1FWD and MUT-TRAP VAL1REV were used to generate pTRAP-**VAL**IIGG and then this plasmid was mutated to **VAL**IIG**V** using primers, MUT-TRAP-VALGV-2FWD and MUT-TRAP-VALGV-2REV. For the TRAP-FFF mutant, first AGGIIGG was mutated to A**FF**IIGG using primers MUT-TRAP-FF1 FWD and MUT-TRAP-FF1 REV to generate pTRAP-AFF and this was used to generate pTRAP-**FFF**IIGG using primers MUT-TRAP-FFF2 FWD and MUT-TRAP-FFF2 REV. Mutagenesis of pTRAP to delete 420 bp of the juxtamembrane region of *TRAP* was performed using primer pairs Juxtamem-MUT1FWD and Juxtamem-MUT2REV to generate pTRAP-JMD. To generate the double mutant containing the juxtamembrane deletion and an altered rhomboid cleavage site, pTRAP-FFF was used to delete 420 bp of the juxtmembrane of TRAP using the same primer pairs. All constructs were sequenced to confirm the presence of the desired mutations.

### Generation of mutant parasites

*P. berghei* ANKA GFP 507clone 1 parasites, which express GFP under the control of the ef1a promoter were used for transfection [37]. Each targeting plasmid was digested with EcoRV and XhoI to liberate the fragment and transfections were performed as previously outlined [37] using 10 µg of digested plasmid DNA and the Amaxa Nucleofector (program U33). Transfected parasites were injected i.v. into Swiss Webster mice and drug resistant parasites were selected using pyrmethamine in the drinking water. Once a parental population was obtained, cloning by limiting dilution was performed in mice [37].

### Diagnostic PCRs

For each clone, integration of the DNA fragment used for transfection at the correct location was confirmed by PCR using 300 ng of genomic DNA isolated from recombinant parasites. To confirm integration at the 5′ end, primers TX-1TRAP5′INT-FWD and 5UTRhDHFRseqREV were used; at the 3′ end, primers hDHFR-3UTRseq and TX-2TRAP3′INT-REV were used; and to verify that there was no contamination with wild type parasites, primers TX-1 TRAP5′INT-FWD and 5′UTRPbTRAP-REV were used. To amplify the TRAP open reading frame, primers SEQPbTRAP2-FWD and SEQPbTRAP3-REV were used and the resulting PCR product was sequenced to confirm the presence of the desired point mutations and/or deletions.

### Antibodies

Monoclonal antibody (mAb) 3D11 directed against the repeat region of *P.berghei* CSP [38] and mAb 2E6 directed against *P.berghei* Hsp70 [39] were used to stain sporozoites and EEFs respectively. UIS-4 polyclonal antiserum, specific for the hepatic stage parasitophorous vacuole [26] and antiserum specific for the cytoplasmic domain of *P. berghei* TRAP [7] were gifts from Dr. Stefan Kappe and Dr. Ali Sultan, respectively. Antiserum to the repeat region of *P.berghei* TRAP was generated in rabbits using the repeat peptide AEPAEPAEPAEPAEPAEPCNH_2_ synthesized and purified by Anaspec Incorporated. The peptide was conjugated to keyhole limpet hemacyanin and the rabbit was immunized and boosted as previously outlined [40]. For generation of polyclonal antisera against the C-terminus of PfROM4, the last 49 amino acids of the protein was amplified from a plasmid containing a synthetic *PfROM4* gene (pHAROM4_synth_ [14]) using forward primer 5′-GGATCCTATAGCC CCCTCGGCCAGATCAAG-3′ and reverse primer 5′-CTCGAGCTTGTTGCAGTAA TACCGAGTGGCTTC-3′, and cloned into pGex4T1 vector (Amersham Bioscience) for protein expression. Protein was purified using the QIAGEN Ni-NTA superflow resin under denaturing conditions according the manufacturer's instructions. Antiserum was raised in rabbits by Eurogentec S.A. according to their standard protocol. IgG fraction of PfROM4 C-terminal antiserum was purified using a Protein G agarose column according the manufacturer's instructions (Pierce).

### Analysis of parasite development in the mosquito

*Anopheles stephensi* mosquitoes were fed on mice infected with recombinant parasite lines once abundant gametocyte stage parasites were observed. Days 14, 16 and 18 post-blood meal, mosquito midguts, hemolymph and salivary glands were harvested for determination of sporozoite numbers, respectively. Although there is some variation among different institutions as to the optimal time to harvest sporozoites, at our facility these are the times when sporozoite numbers in each respective compartment are at their maximum. For midgut and salivary gland sporozoite counts, organs from 10 to 15 mosquitoes were pooled and homogenized and released sporozoites were counted using a hemocytometer. For hemolymph sporozoite counts, the hemocoel was perfused with DMEM and the first two drops of perfusate collected from 10 to 15 mosquitoes was pooled and sporozoites were counted as above. To determine the proportion of salivary gland sporozoites that were inside the glands, day 18 salivary glands were dissected, incubated with 50 µg/ml trypsin for 15 min at 37°C and centrifuged at 100× g for 5 min at 4°C. The supernatant was collected and the salivary glands were homogenized to release internalized sporozoites and the sporozoites in each compartment were counted using a hemocytometer.

### Metabolic labeling

Experiments with wild type sporozoites were performed with 10^6^ sporozoites per condition whereas experiments with recombinant sporozoites (TRAP-rWT, TRAP-VAL and TRAP-FFF) were performed with 2.5×10^5^ sporozoites. Sporozoites were metabolically labeled in DMEM without Cys/Met, containing 0.2% BSA, and 400 µCi/ml [^35^S]-Cys,Met for 1 hour at 28°C and then kept on ice or chased in DMEM with Cys/Met and 0.2% BSA for 2 hours at 28°C in the absence or presence of the indicated protease inhibitors. The concentrations of inhibitors used were: 100 µM TLCK, 75 µM leupeptin, 0.3 µM aprotinin, 100 µM 3,4 DCI, 5 mM EDTA, 10 µM E64, 1 mM PMSF, and 1 µM pepstatin. Metabolically labeled and chased sporozoites were spun at 16,000× g for 4 min, and the pellets and supernatants were separated. The sporozoite pellets were lysed in SDS/Urea lysis buffer [1% SDS, 4 M Urea, 150 mM NaCl, 50 mM Tris-HCl pH 8.0, 1X Protease Inhibitor Cocktail, (Roche)] for 1 hr at 4°C and TRAP was immunoprecipitated with TRAP repeat antiserum or TRAP C-terminal antiserum conjugated to Protein A agarose beads overnight at 4°C. The beads were then washed with lysis buffer (1% Triton X-100, 150 mM NaCl, 50 mM Tris-HCl, pH 8.0) followed by lysis buffer containing 500 mM NaCl and pre-elution buffer (0.5% Triton X-100, 10 mM Tris-HCl, pH 6.8). TRAP was eluted with 1% SDS in 0.1 M glycine, pH 1.8, neutralized with 1.5 M Tris-HCl, pH 8.8, and run on a 7.5% SDS–polyacrylamide gel under non-reducing conditions using 18×16 cm gels (Hoefer SE600 system). Gels were fixed, enhanced with Amplify (GE Biosciences), dried and exposed to film.

### Immunoblot of sporozoite lysates

Experiments with TRAP-FFF and TRAP-VAL mutants used salivary gland sporozoites whereas those with TRAP-JMD and TRAP-DMut mutants utilized midgut sporozoites due to the low numbers of salivary gland sporozoites in the TRAP-DMut parasite. Sporozoites were lysed in 6X SDS-PAGE sample buffer and 3×10^4^ sporozoite equivalents were loaded per lane of a 18×16 cm, 7.5% SDS-polyacrylamide gel and separated under non-reducing conditions. The proteins were then transferred to a PVDF membrane, and incubated with either TRAP repeat antiserum (1∶100) or mAb 3D11 (4 µg/ml), followed by either anti–rabbit (1∶50,000) or anti–mouse Ig (1∶200,000) conjugated to HRP. Bound antibodies were visualized using the enhanced chemiluminescence detection system (GE Biosciences). Western blot analysis for PbROM4 expression was performed by loading lysates of Nycodens purified *P. berghei* asexual blood stage schizonts and dissected *P. berghei* wildtype sporozoites on an SDS-polyacrylamide gel, proteins were transferred as outlined above and the blot was incubated with anti-ROM4 antisera and developed as above. PbROM4 conditional knockouts were used to demonstrate the specificity of this antisera. These parasites were generated by a double crossover strategy positioning the transactivator under control of the endogenous *P. berghei* ROM4 promoter, while PbROM4 expression was controlled by the inducible tet-operator containing promoter, resulting in an inducible copy of PbROM4. Several independent transgenic parasite pools were obtained, cloned and verified by PCR (P. Pino and D. Soldati-Favre, unpublished data). Asexual stage transgenic parasites were inoculated into mice which were treated or not with anhydrotetracycline (ATc) in the drinking water for 36 hrs, parasites were collected and allowed to mature to schizonts in vitro for 12 hrs in the presence or absence of ATc, schizonts were purified and lysates were evaluated by western blot as outlined above.

### Immunofluorescence assays

Wild type or mutant salivary gland sporozoites were centrifuged onto glass 8-chambered Lab-Tek wells at 300× g for 2 min at 12°C, and then fixed with 4% PFA for 1 hr at RT. For total TRAP staining, sporozoites were also permeabilized with cold methanol for 15 min at −20°C following fixation with PFA. Sporozoites were stained with TRAP repeat antiserum (1∶100) diluted in 1% BSA and 5% goat serum in PBS for 1 hr at 37°C. Following this, wells were incubated with anti-rabbit IgG Alexa Fluor 594 for 1 hr at 37°C and coverslips were then mounted in Citifluor (Ted Pella). Sporozoites were visualized by phase and fluorescence microscopy with a Nikon 100X PlanApo objective on a Nikon E600. Images for quantitative analysis were acquired using a DS-Ri1 digital camera with identical resolution, gain and color settings in which parasites were exposed for 75 ms in the fluorescence channel. Intensity measurements were calculated using the NIS Elements Br 3.2 software (Nikon) and statistical analysis was performed using the Student's unpaired t-test.

### Gliding motility assay

Glass 8-chambered Lab-tek wells were coated with 10 µg/ml mAb 3D11 in TBS overnight at 25°C. Salivary gland sporozoites were dissected in 3% BSA/DMEM and 2×10^4^ sporozoites were added to each well and incubated for 1 hr at 37°C. Wells were then fixed in 4% PFA and stained with biotinylated 3D11 for 1 hr at 25°C, followed by incubation with Streptavidin-FITC (1∶100, GE Biosciences) for 1 hr at 25°C. Slides were mounted using Citifluor mounting medium and visualized as above. The number of sporozoites with and without trails was counted. Assays in which TRAP was visualized in the trails were performed in the same way except that Lab-tek wells were not precoated with antibody and trails were visualized using TRAP repeat antiserum followed by anti-rabbit IgG conjugated to FITC. For experiments with protease inhibitors, the sporozoites were pre-incubated with indicated inhibitor for 1 hr at 28°C, and then added to the wells in the continued presence of the inhibitor. The concentrations of inhibitors used were: 100 µM TLCK, 75 µM leupeptin, 100 µM 3,4 DCI, 10 µM E64, 1 mM PMSF, and 1 µM pepstatin.

### Live imaging of sporozoite motility

10^4^ salivary gland sporozoites were dissected in 3% BSA/DMEM, spun at 16,000× g for 4 min, and the pellets were resuspended in 3% BSA/DMEM at 4°C for 1 hr. Sporozoites were incubated at 37°C for 5 min before being added to a 14 mm glass bottom dish (MatTek) and then visualized using a Zeiss LSM 510 confocal microscope or Leica Inverted Laser Scanning Confocal Microscope (Model Number TCS SP2 AOBS), with a stage heated to 37°C. Image files were processed using Leica LCS software and Image J. Manual tracking was performed using the Image J Manual Tracker plug-in, and the data was compiled in Microsoft Excel and Sigma Plot.

### Cell traversal assay

2×10^4^ salivary gland sporozoites in 1%BSA/DMEM were added to the monolayers of Hepa 1,6 cells in the presence of 1 mg/ml TOTO-1 (Invitrogen), a dimeric cyanine nucleic acid dye, for 1 hr at 37°C. Cells were washed with DMEM, fixed in 4% PFA and the number of TOTO-1 positive cells in 50 fields was counted. When indicated, sporozoites were pre-incubated with 1 mM cytochalasin D for 10 min at 28°C and added to cells in the continued presence of the compound.

### Invasion and development assays

5×10^4^ salivary gland sporozoites were added to coverslips of semi-confluent Hepa 1–6 cells in DMEM with 10% fetal calf serum and 0.1 mM glutatmine (DMEM/FCS) for 1 hr at 37°C and cells were then fixed with 4% PFA and stained with a double staining technique that distinguishes extracellular from intracellular sporozoites [41], [42]. To determine the kinetics of cell entry, sporozoites were added to cells as outlined above and fixed with 4% PFA at 15, 30 or 45 minutes after their addition. Sporozoites were stained with the double staining assay and those sporozoites that were in the process of entering, i.e. half stained as an intracellular sporozoite and half stained as an extracellular sporozoite, were counted and compared to the total number of sporozoites. To assess productive invasion, sporozoites were incubated with cells for 6 hrs at 37°C, washed, fixed with methanol and stained with UIS-4 antiserum (1∶500 dilution) and mAb 3D11 (1 µg/ml). To quantify EEF development, cells with sporozoites were grown for 40 hrs after which they were fixed with methanol and stained with mAb 2E6 followed by goat anti-mouse IgG conjugated to rhodamine.

### Determination of pre-patent period

Swiss Webster or C57BL/6 mice were injected with the indicated number of salivary gland sporozoites either by i.v. or i.d. inoculation. The onset of blood stage infection was determined by observation of Giemsa-stained blood smears beginning on the third day after sporozoite inoculation.

## Supporting Information

Figure S1 **Generation of** ***TRAP*** **mutants.** (A) Targeting strategy for replacement of the endogenous *TRAP* locus with wild type or mutant *TRAP*. The transfection plasmid, pTRAP contains 500 bp of TRAP 5′UTR (red line), the selectable marker hDHFR (black box) with its upstream and downstream control elements (black lines), and the *TRAP* gene (red box) flanked by its upstream and downstream control elements (red lines). The dashed black lines indicate the location of homologous recombination with the endogenous *TRAP* locus. (B) Diagnostic PCRs were used to verify successful recombination and the presence of the desired mutations. Primers A and B were used verify integration at the 5′ end. Primers C and E were used to verify integration at the 3′ end. Primers A and D were used to verify the absence of WT untransfected genomic DNA. Primers F and G (shown in panel A) were used to amplify the *TRAP* open reading frame for sequencing to confirm the presence of the desired mutations. Restriction sites are abbreviated as follows: H3, HindIII; E1, EcoRI; EV, EcoRV; Xho, XhoI; Kpn, KpnI. Primer sequences can be found in Table S1.
(PDF)

Figure S2 **Primary structure of** ***Plasmodium berghei*** **TRAP and TRAP-JMD.** Full-length TRAP has a signal sequence that is predicted to be cleaved after amino acid residue 24 (http://www.cbs.dtu.dk/services/SignalP), followed by a predicted A-domain (magenta), a region with similarity to the type I thrombospondin repeat (green) and a repeat region. The juxtamembrane region deleted in the TRAP-JMD mutant is in italics and underlined. In blue is the predicted transmembrane domain with the putative rhomboid cleavage site shown in bold.
(DOC)

Figure S3 **TRAP trails of gliding TRAP-mutants.** TRAP mutants were allowed to glide on glass slides for 1 hr at 37°C and then slides were fixed and stained with anti-TRAP repeat antisera followed by a secondary conjugated to a fluorophore. Shown are representative images except in the case of TRAP-DMut where we show one of the two trails we found.
(EPS)

Figure S4 **Confirmation of ROM4 antiserum specificity.** Two independent clones of PbROM4 conditional knockout parasites were grown in mice given anhydrotetracycline (ATc) in their drinking water (+) or not (−), schizonts were purified and proteins separated by SDS-PAGE. The presence of HA-tagged PbROM4 was determined by Western blot using anti-ROM4 C-terminal IgG and profilin (PRF) was used as a loading control. The positions of molecular weight markers are shown on the left.
(EPS)

Table S1 **Sequence of primers used in this study.**
(DOC)

Video S1 **Movie of TRAP-rWT sporozoites gliding.**
(MP4)

Video S2 **Movie of TRAP-VAL sporozoites gliding.**
(MP4)

Video S3 **Movie of TRAP-FFF sporozoites gliding.**
(MP4)
