# Supplementary figures and images for: Shedding of TRAP by a Rhomboid Protease from the Malaria Sporozoite Surface Is Essential for Gliding Motility and Sporozoite Infectivity
Source: PLoS Pathog. 2012 Jul 26;8(7):e1002725. doi: 10.1371/journal.ppat.1002725 (PMC3406075; doi:10.1371/journal.ppat.1002725)

A

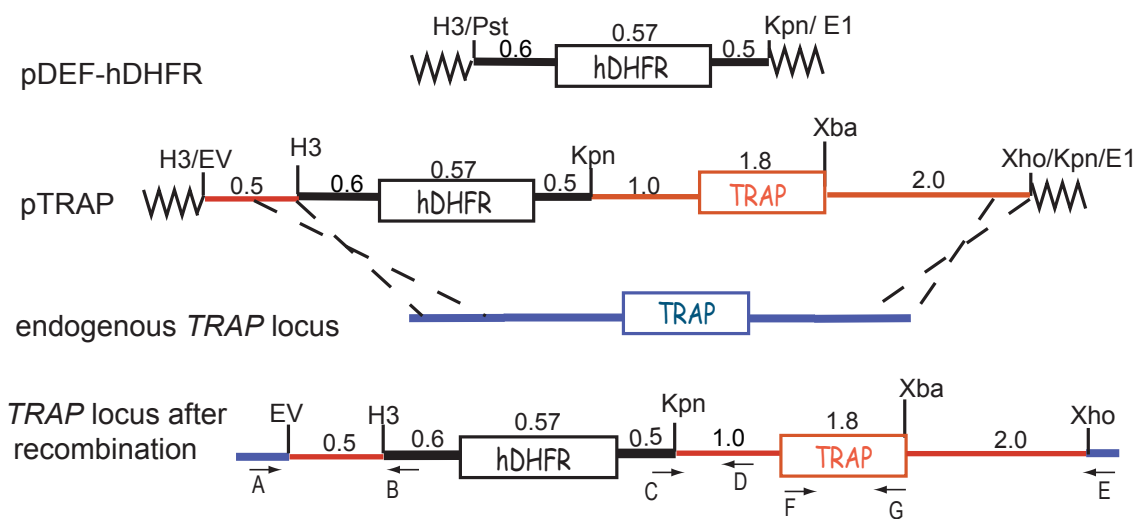

B

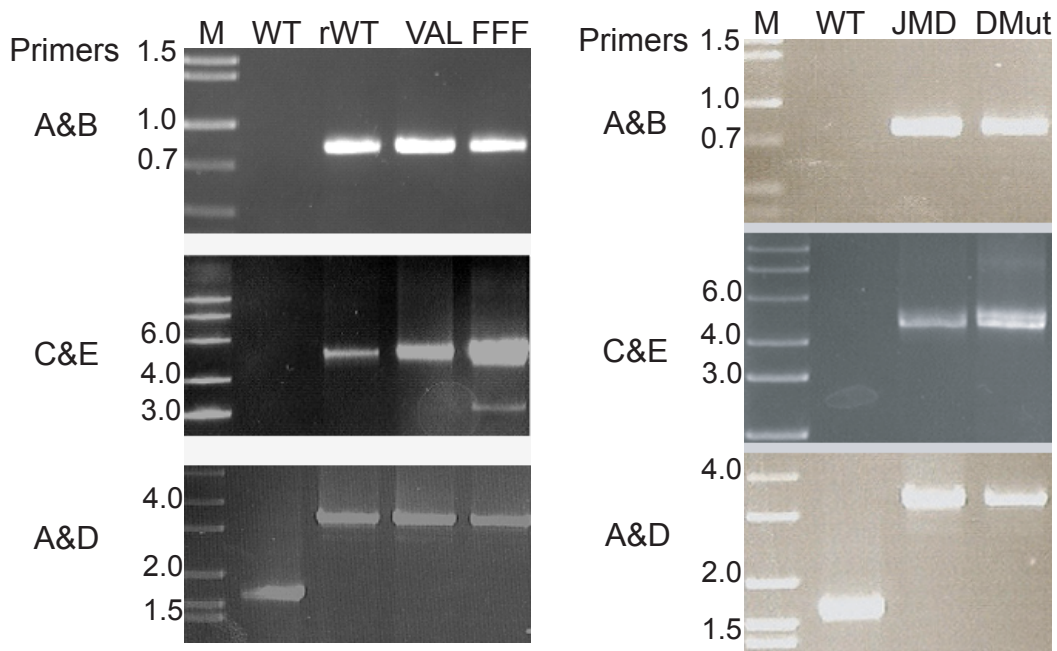

Supplement: Figure S1 — Generation of TRAP mutants. (A) Targeting strategy for replacement of the endogenous TRAP locus with wild type or mutant TRAP. The transfection plasmid, pTRAP contains 500 bp of TRAP 5′UTR (red line), the selectable marker hDHFR (black box) with its upstream and downstream control elements (black lines), and the TRAP gene (red box) flanked by its upstream and downstream control elements (red lines). The dashed black lines indicate the location of homologous recombination with the endogenous TRAP locus. (B) Diagnostic PCRs were used to verify successful recombination and the presence of the desired mutations. Primers A and B were used verify integration at the 5′ end. Primers C and E were used to verify integration at the 3′ end. Primers A and D were used to verify the absence of WT untransfected genomic DNA. Primers F and G (shown in panel A) were used to amplify the TRAP open reading frame for sequencing to confirm the presence of the desired mutations. Restriction sites are abbreviated as follows: H3, HindIII; E1, EcoRI; EV, EcoRV; Xho, XhoI; Kpn, KpnI. Primer sequences can be found in Table S1. (PDF) [file ppat.1002725.s001.pdf]
