# Supplementary material for: Shedding of TRAP by a Rhomboid Protease from the Malaria Sporozoite Surface Is Essential for Gliding Motility and Sporozoite Infectivity
Source: PLoS Pathog. 2012 Jul 26;8(7):e1002725. doi: 10.1371/journal.ppat.1002725 (PMC3406075; doi:10.1371/journal.ppat.1002725)
Supplement: Figure S2 — Primary structure of Plasmodium berghei TRAP and TRAP-JMD. Full-length TRAP has a signal sequence that is predicted to be cleaved after amino acid residue 24 (http://www.cbs.dtu.dk/services/SignalP), followed by a predicted A-domain (magenta), a region with similarity to the type I thrombospondin repeat (green) and a repeat region. The juxtamembrane region deleted in the TRAP-JMD mutant is in italics and underlined. In blue is the predicted transmembrane domain with the putative rhomboid cleavage site shown in bold. (DOC) [file ppat.1002725.s002.doc]

Figure S2

*Plasmodium berghei* TRAP

MKLLGNSKYFFVVLLLCISVFLNGQEILDEIKYSEEVCNEQIDLHILLDGSGSIGHSNWISHVIPMLTTLVDNLNISRDEINISMTLFSTYARELVRLKRYGSTSKASLRFIIAQLQNNYSPHGTTNLTSALLNVDNLIQKKMNRPNAIQLVIILTDGIPNNLKKSTTVVNQLKKKDVNVAIIGVGAGVNNMFNRILVGCGKLGPCPYYSYGSWDQAQTMIKPFLSKVCQEVEKVALCGKWEEWSECSTTCDNGTKIRKRKVLHPNCAGEMTAPCKVRDCPPKPVAPPVIPIKVPDVPVKPVEPIEPAEPAEPAEPAEPAEPAEPAEPAEPAEPAEPAEPAEPAEPAEPAEPAEPAEPAEPAEPAKPAEPAEPAEPAEPAEPVNPDNPILP*IKPEEPSGGAEPLNPEVENPFIIPDEPIEPIIAPGAVPDKPIIPEESNELPNNLPESPSDSQVEYPRPNDNGDNSNNTINSNKNIPNKHVPPTDDNPYKGQEERIPKPHRSNDEYIYYNNANNNDKLEPEIPSKDYEENK*SKKQSKSNNGYKI**AGGIIGG**LAIIGCIGVGYNFIAGSSAAAMAGEAAPFEDVMADDEKGIVENEQFKLPEDNDWN
