# Supplementary material for: Shedding of TRAP by a Rhomboid Protease from the Malaria Sporozoite Surface Is Essential for Gliding Motility and Sporozoite Infectivity
Source: PLoS Pathog. 2012 Jul 26;8(7):e1002725. doi: 10.1371/journal.ppat.1002725 (PMC3406075; doi:10.1371/journal.ppat.1002725)
Supplement: Table S1 — Sequence of primers used in this study. (DOC) [file ppat.1002725.s005.doc]

**Table S1: Primers Used for Generation of Targeting Plasmids**

|  | | |
| --- | --- | --- |
| **Generation of pTRAP** | |  |
| PbTRAP5’UTR-FWD | 5’CAGCTAAGCTTGATATCCAAAAGTACAACATAAAAGGAACTGAATG-3’  HindIII site is underlined and EcoRV site is double underlined | |
| PbTRAP5’UTR-REV | 5’- CAGCTAAGCTTCTCTTTTTTACATGTATTCAATTAGCATGC -3’  HindIII site is underlined | |
| PbTRAP FWD-KpnI | 5’CTGGTACCGAGAAATTCCTTCGTTTTATAATTTATAAAGC- 3’  KpnI site is underlined | |
| PbTRAP REV- XhoI/KpnI | 5’CCGGTACCCTC GAGCAGAGCCAGGATATGAACTATTTTAAAAC-3’  KpnI site is underlined and XhoI site is double underlined | |
|  |  | |
| **Generation of pTRAP-VAL** | | |
| MUT-TRAP-VAL 1 FWD | 5’CTAATAACGGATATAAAATTGTTGCTCTTATTATTGGAGGATTAGC 3’ | |
| MUT-TRAP VAL1 REV | 5’GCTAATCCTCCAATAATAAGAGCAACAATTTTATATCCGTTATTAG -3’ | |
| MUT-TRAP VALGV-2FWD | 5’ATTGTTGCTCTTATTATTGGAGTTTTGCTATAATTGGATGCATAGGTGTTG-3’ | |
| MUT-TRAP-VALGV-2REV | 5’-CAACACCTATGCATCC AATTATAGCTAAAACTCCAATAATAAGAGCAACAAT- 3’ | |
|  |  | |
| **Generation of pTRAP-FFF** | | |
| MUT-TRAP-FF1 FWD | 5’GCAGTCTAAATCTAATAACGGATATAAAATTGCTTTTTTTATTAT TGGAGGATTAGCTATAATTGGATG 3’ | |
| MUT-TRAP-FF1 REV | 5’CATCCAATTATAGCTA ATCCTCCAATAATAAAAAAAGCAATTT TATATCCGTTATTA GATTTAGACTGC-3’ | |
| MUT-TRAP FFF2 FWD | 5’ GCAGTCTAAATCTAATAACGGATATAAAATTTTTTTTTTTATTA TTGGAGGATTA GCTATAATTGGATG- 3’ | |
| MUT-TRAP-FFF2 REV | 5’- CATCCAATTATAGCTAATCCTCCAATAATAAAAAAAAAAATTT TATATCCGTTATTAGATTTAGACTGC -3’ | |
|  |  | |
| **Generation of pTRAP-JMD/DMUT** | | |
| Juxtamem-MUT1FWD | 5’- CCAGTCAACCCAGATAATC CAATTTTACCAAGTAAAAAGCA GTCTAAATCTAATAACGG-3’ | |
| Juxtamem-MUT2REV | 5’- CCGTTATTAGATTTAGACTGCTTTTTA CTTGGTAAAATTGGATT ATCTGGGTTGACTGG-3” | |
|  |  | |
| **Diagnostic PCRs*** | | |
| (A) TX-1 TRAP 5’INT-FWD | 5’-CGATCAAATTAAAGAGCAAAAGTTCAC-3’ | |
| (B) 5UTR hDHFRseqREV | 5’-CTTTGAGG GGTGAGCATTTAAAGC-3’ | |
| (C) hDHFR-3UTRseq | 5’- GTCTCTTCAATGATTCATAAATAGTTGG-3’ | |
| (D)TX-2TRAP3’INT-REV | 5’-CAATCCAATAAGGATCCTAATAATTATTC-3’ | |
| (E)5’UTRPbTRAP-REV | 5’-GCTAGCAGGATCCTATAAGGGAAAGGGAAAATGGGC-3’ | |
| (F)SEQPbTRAP2-FWD | 5’-CAATTCGTATTATATGCGTATGTG-3’ | |
| (G)SEQPbTRAP3-REV | 5’- GTTCCAGTCATTATCTTCAGG-3’ | |
|  |  | |

*letters before the primer names refer to the letters shown in Supplementary Figure 1.
